# Natural anti-inflammatory agents for pain relief

**DOI:** 10.4103/2152-7806.73804

**Published:** 2010-12-13

**Authors:** Joseph C. Maroon, Jeffrey W. Bost, Adara Maroon

**Affiliations:** Department of Neurosurgery, University of Pittsburgh Medical Center, Pittsburgh, PA, USA; 1 Vanderbilt University, Nashville, TN, USA

**Keywords:** Alternative treatments, inflammation, natural anti-inflammatories, pain

## Abstract

The use of both over-the-counter and prescription nonsteroidal medications is frequently recommended in a typical neurosurgical practice. But persistent long-term use safety concerns must be considered when prescribing these medications for chronic and degenerative pain conditions. This article is a literature review of the biochemical pathways of inflammatory pain, the potentially serious side effects of nonsteroidal drugs and commonly used and clinically studied natural alternative anti-inflammatory supplements. Although nonsteroidal medications can be effective, herbs and dietary supplements may offer a safer, and often an effective, alternative treatment for pain relief, especially for long-term use.

## INTRODUCTION

Pain, heat, redness, and swelling (dolor, calor, rubor, tumor) are the classic manifestations of the inflammatory process. Abnormalities of the joints of the spine, associated muscles, tendons, ligaments and bone structural abnormalities can all result in pain and need for neurosurgical consultations. Typically, patients will not require immediate surgical intervention, and therefore require treatments to reduce pain and enhance quality of life activities.[71]

In most cases, the genesis of pain is inflammatory, regardless of the etiology. With the elucidation of the role of inflammatory cytokines, there is now a clear understanding of the pathways by which many anti-inflammatory drugs can alleviate inflammation and relieve pain.

The use of non-steroidal anti-inflammatory drug (NSAID) medication is still the mainstay of most classically taught clinicians for joint and spine related inflammatory pain, despite their commonly known side effects [Table 1]. NSAID mechanisms are primarily through interaction with proinflammatory cytokines interleukin (IL)-1a, IL-1b, IL-6 and tumor necrosis factor (TNF-α). Increased concentrations of TNF-α are believed to cause the cardinal signs of inflammation to occur.[44]

**Table 1:** The commonly known and documented side effects of steroid-based medications[106]

Side effects of steroid-based medications	
Increased risk of infection	Impaired wound healing
Dermatitis	Increased appetite
Fluid retention edema	Weight gain
Fat deposits in face, chest, upper back and stomach	Worsening of previously acquired medical conditions
Mood change	Depression
Hypertension	Hyperglycemia
Cushingoid-like state	Adrenal suppression and crisis
Stomach ulcers	Cataracts
Osteoporosis	

**Figure 1:**
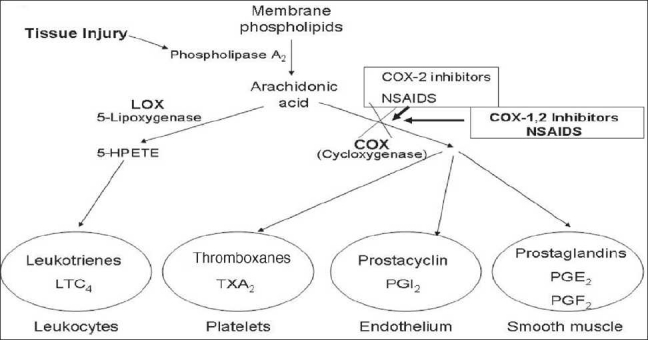
Schematic showing that when a cell membrane is injured the arachidonic acid pathway is activated to initiate the local inflammatory response through the production of prostaglandins, thromboxanes, and leukotrienes. Their activation requires the enzymes COX and LOX. The NSAIDs can block COX action and thereby prevent the formation of the COX-derived inflammatory mediators. 5-HPETE = 5-hydroperoxyeicosatetraenoic acid; LTC4 = leukotriene C4; PGE2 = prostaglandin E2; PGF_2_ = prostaglandin F2; PGI_2_ = prostacyclin; TXA_2_ = thromboxane.

These proinflammatory cytokines result in chemoattractant for neutrophils and help them to stick to the endothelial cells for migration. They also stimulate white cell phagocytosis and the production of inflammatory lipid prostaglandin E2 (PGE_2_). NSAIDs’ ability to interfere with the production of prostaglandin during the inflammatory cascade is the major mechanism cited for the anti-inflammatory success of these medications [Figure 1].[112]

## INFLAMMATORY PATHWAYS

Prostaglandins act as short-lived localized hormones that can be released by any cell of the body during tissue, chemical, or traumatic injury, and can induce fever, inflammation, and pain, once they are present in the intercellular space. Thromboxanes, which are also hormone activators, can regulate blood vessel tone, platelet aggregation, and clot formation to increase the inflammatory response.[92,82] The inflammatory pathway is a complex biochemical pathway which, once stimulated by injury, leads to the production of these and other inflammatory mediators whose initial effect is pain and tissue destruction, followed by healing and recovery.[34,51] A major component of the inflammatory pathway is called the arachidonic acid pathway because arachidonic acid is immediately released from traumatized cellular membranes. Membrane-based arachidonic acid is transformed into prostaglandins and thromboxanes partly through the enzymatic action of cyclooxygenase (COX)[34,57]. There are two types of COX enzymes, COX-1 and COX-2. Both the enzymes act similarly, but selective inhibition (as accomplished by selective COX-2 inhibiting NSAIDs) can make a difference in terms of side effects.

Acetylsalicylic acid works by irreversibly disabling the COX enzymes to block the cascade [Figure 1]. NSAIDs have evolved from blocking both COX-1 and COX-2 to selectively only blocking COX-2 in order to inhibit the inflammatory response and reduce the production of inflammatory prostaglandins and thromboxanes. The major push to develop the selective COX-2 inhibitors has been the recognition of significant complications associated with the nonselective COX-1 and COX-2 NSAIDs. Nonselective NSAIDs’ major side effects include significant gastrointestinal upset, gastritis, ulceration, hemorrhage, and even death. By locking COX-1, which also normally acts to protect the gastrointestinal mucosa, nonselective NSAIDs and aspirin can cause significant gastric tissue damage.[34,51,78,91,3,101,115]

Various studies have also shown that NSAIDs can delay muscle regeneration and may reduce ligament, tendon, and cartilage healing.[4,13,77] Specifically, NSAIDs are believed to wipe out the entire inflammatory mediated proliferative phase of healing associated with WBC actions (days 0–4). A study of the effects of NSAIDs on acute hamstring injuries was done in humans by Reynolds *et al*.,[93] and these investigators concluded that patients who used NSAIDs did not experience a greater reduction of pain and soft-tissue swelling when compared with the placebo group. Interestingly enough, the authors noted that the NSAIDs’ group had worse pain associated with severe injuries compared with the placebo group.

The NSAIDs are also known to have adverse effects on kidney function.[31] Dehydration or preexisting chronic renal failure or disease, resulting in stimulation of the renin–angiotensin system, may predispose certain populations to acute renal failure through inhibition of prostaglandin synthesis, which can occur when taking NSAIDs.[31] The National Kidney Foundation asserts that approximately 10% of kidney failures per year are directly correlated to substantial overuse of NSAIDs.

### Life-threatening side effects of selective COX-2 NSAIDs

in December 1998, celecoxib (Celebrex) was approved by the Food and Drug Administration (FDA) as the first selective COX-2 inhibitor for treatment of arthritis pain.[92,13,22] Rofecoxib (Vioxx) was approved several months later, followed by valdecoxib (Bextra).[92,28,67,79] These NSAIDs were designed to allow continued production of the gastrointestinally protective prostaglandins produced through the COX-1 enzyme system while blocking the COX-2 enzyme that produces the inflammatory prostaglandins.[34,45,51,89]

Celebrex, Vioxx, and Bextra quickly became the mainstay for the treatment of chronic pain conditions related to inflammation.[71] Within a few years, an estimated 15–20 million people in the US were using selective COX-2–inhibiting NSAIDs on a long-term basis. These drugs became the most commonly used pharmaceutical agent with more than 70 million NSAID prescriptions written each year and 30 billion over-the-counter NSAID tablets sold annually. It was estimated that 5–10% of the adult population used NSAIDs, and among the elderly (a group at higher risk of nonselective NSAID-induced gastrointestinal complications), the use of these drugs was as high as 15%. The general acceptance of these drugs was due to the perceived lack of serious gastrointestinal side effects that had been associated with the nonselective class of NSAIDs.[26,119]

On September 30, 2004, Merck Research Laboratories announced the global withdrawal of rofecoxib (Vioxx), its primary selective COX-2–inhibiting NSAID.[52,90,122] Analysis of the results of the Adenomatous Polyps Prevention on Vioxx study (known as the APPROVe study) showed that there was double the risk of serious thromboembolic events, including myocardial infarction, which became apparent after 18 months of Vioxx treatment.[26] Selective COX-2 NSAID’s thrombotic mechanism of action is based on COX-1’s unopposed action to continued platelet synthesis of thromboxane. Thromboxane is a thrombogenic and atherogenic eicosanoid. Prostacyclin prevents formation of platelet clotting. By inhibiting COX-2 that blocks production of prostacyclin (PGI2) there is unopposed thromboxane which will increase the clotting risk. Thus, inhibiting prostacyclin led to the increased risk of thrombotic cardiovascular and cerebrovascular events.[5,26,73,123]

### Natural compounds for inflammation

Because of the significant side effect profiles of steroidal and NSAID medications, there is a greater interest in natural compounds, such as dietary supplement and herbal remedies, which have been used for centuries to reduce pain and inflammation.[94] Many of these natural compounds also work by inhibiting the inflammatory pathways in a similar manner as NSAIDs. In addition to the COX pathway, many natural compounds act to inhibit nuclear factor-kB (NF-kB) inflammatory pathways.

### NF-kB inflammatory pathways and cytokines

**Figure 2:**
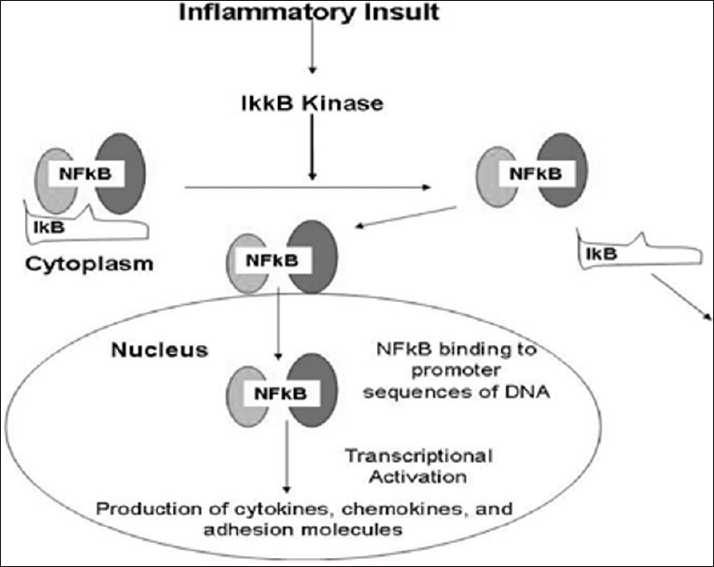
Schematic showing another inflammatory pathway that is activated by tissue injury. This is the NF-kB activation, in which once the protein is free as a result of tissue injury, it can enter the cell nucleus and activate the DNA to enhance the inflammatory response further by the production of additional cytokines, chemokines, and adhesion molecules (IKKB = IkB kinase)

The NF-kB molecule is a transcription factor that controls the transcription of DNA for the perpetuation of the inflammatory immune response. It acts as a switch to turn inflammation on and off in the body. NF-kB has the ability to detect noxious stimuli, such as infectious agents, free radicals, and other cellular injuries, and then directs DNA to produce inflammatory cytokines. The NF-kB proteins are localized in the cytoplasm of the cell and are associated with a family of inhibitory proteins known as inhibitor of kB (IkB).[43,119] The TNF-α, and especially IL-1b, can also directly stimulate enzymes known as matrix metalloproteinases, which break down extracellular collagen matrix, a hallmark of inflammatory joint disease.[32,76,77] The IkB proteins are normally bound to NF-kB and block their nuclear localization signal. A variety of provoking stimuli can degrade the IkB and result in the nuclear translocation of NF-kB to be free to activate DNA synthesis of inflammatory cytokines [Figure 2].

Aspirin is now believed to target both the NF-kB and COX pathways. These agents inhibit the NF-kB pathway in endothelial cells and block NF-kB activation to inhibit leukocyte recruitment.[114,115,116] NSAIDs have also been found to inhibit both the COX system and the NF-kB pathway. Immunosuppressant drugs also reduce nuclear expression of NF-kB.[39,70,75] Research now indicates that blocking the activation of NF-kB along with other inflammation mediators [Table 2] is the major mechanism for reducing inflammation by natural compounds.

**Table 2:** Several examples of inflammation triggering factors, pathway mediators and conditions modulated by natural compounds

Natural compounds and inflammatory pathway modulation	
Inflammation triggers	
Stress	Infection
Radiation	Allergic immune response
Trauma/injury	Arachidonic acid
Inflammatory diet	
Inflammatory pathways	
NF-Kβ	Leukotrienes
IL-1,6	NO
CRP	Lipoxygenase
COX-1 and -2	TNF-α
Prostaglandins	Adhesion molecules
Thromboxanes	Reactive oxygen species (ROS)
Collagenase/MVP	Cytokines
Conditions modulated	
Pain	Atherosclerosis
Inflammation	Thrombosis
Insulin resistance	Autoimmune response
Cancer	Neurodegeneration

### Examples of natural anti-inflammatory

Plant- and animal-derived nutraceutical preparations have been used for hundreds and even thousands of years to obtain effective pain relief. Herbal medications are becoming increasingly popular because of their relatively few side effects. Nevertheless, there are problems associated with these dietary supplements, and their use requires knowledge of their biological action, clinical studies (both affirmative and negative), and potential interactions with other nutraceutical products and prescription medications.

The evaluation of nutraceutical preparations with appropriately designed controlled studies has exploded in recent years. There is now a greater degree of confidence based on controlled study design and improved quality of the investigators that has strengthened positive findings found using natural compounds to treat diseases. It is important for healthcare practitioners to learn about these scientific studies to counsel patients who are taking various dietary supplements, herbs minerals and vitamins for both disease treatment and prevention.

### Quality considerations

The processes used to prepare herb-derived compounds pose complications when it comes to determining the quantity and concentration of the products.[30,63,102] The preparation processes are not standardized, and therefore, the extraction process and the type of plant used may affect the true concentration of the product. In addition, there is a lack of uniformity within and between manufacturers. Although dietary supplements are not held to the same rigorous testing and standards as pharmaceutically derived medications in the US, there are many regulations that still control their manufacture because these are food products.

The US governmental agencies, through the FDA and others, routinely inspect the manufacture of vitamins or supplements made in this country, as they do for any other food product.[30,63,74] Contaminants, such as the recently discovered high lead content found in various Ayurvedic preparations that were made by an Indian manufacturer and imported into the US,[30,61,63,74,102] are generally thought to be uncommon, but can be a concern when purchasing imported supplements.

Some manufacturers inflate nutraceutical products’ claims and may not cite possible side effects and potential drug interactions. Bleeding complications are associated with white willow bark, ginger, garlic, and others. Therefore, such medicinal preparations are not without risk. Products such as omega-3 essential fatty acids (EFAs) (O3) do have strong scientific support to be considered as an alternative and/or complementary agent to NSAIDs. Published studies have shown the effectiveness of O3 to successfully treat spine-related pain.[71] Capsaicin, oil of camphor, and other natural topical preparations are commonly used for muscle soreness and local application for painful traumatic injuries.[12,16,80] The subsequent sections will review many of these products and discuss both their efficacy and safety issues. As with any drug or natural compounds, additional caution should be used when considering these treatments for children, pregnant or lactating mothers or any other clinical or disease condition that could increase possible risk of side effect or complication.

### Omega-3 EFAs (fish oil)

The use of fish oil (in the form of cod liver oil), an omega-3 EFA, for the treatment of muscular, skeletal, and discogenic diseases, can be traced back to the late 18^th^ century as detailed by Curtis *et al*.,[24,25] Unfortunately, because of the rapid onset of rancidity of this polyunsaturated oil when exposed to air, and hence its disconcerting odor, cod liver oil fell out of favor. With improved extraction techniques, such as using a protective nitrogen blanket and enhanced oxygen-free encapsulation methods, there is less chance of oxidation during the manufacturing process. The therapeutic benefits of fish oil can now be realized without the regurgitation and odor of previous products caused by peroxides and rancid tasting fish oil.[14]

Research has shown that the omega-3 polyunsaturated fatty acids are some of the most effective natural anti-inflammatory agents available.[12,23–25,27,50,85] With the discovery that vascular inflammation is the underlying cause of coronary artery disease, fish and fish oil supplements are now recommended by the American Heart Association for the prevention of this disease.[12,23–25,27,50,85] Countries that have the highest fish consumption also have a lower incidence of neurodegenerative disease and depression.[12,23–25,27,50,85] The biological basis for the effectiveness of fish oil in treating arthritis has been well documented with many positive clinical studies, when compared to traditional pharmaceutical anti-inflammatory agents.[12,23–25,27,50,85]

The active ingredients in fish oil, eicosapentaenoic acid (EPA) and docosahexaenoic acid (DHA), enhance the conversion of COX to prostaglandin E3. A natural anti-inflammatory agent, prostaglandin E3 competitively inhibits the effects of the arachidonic acid conversion to prostaglandin E2, a highly inflammatory substance. Prostaglandin E3 also inhibits the synthesis of TNF-α and IL-1b, both of which are inflammatory cytokines. The EPA and DHA can inhibit the 5-LOX pathway, which converts arachidonic acid to inflammatory leukotrienes, by competitive inhibition as well. When EPA and DHA are incorporated into articular cartridge chondrocyte cell membranes, there is a dose-dependent decrease in the expression and activity of the proteoglycan-degrading aggrecanase enzymes.[12,23–25,27,50,85]

Omega-3 EFA, found in fish oil, can directly reduce the degenerative enzymes, aggrecanase and matrix metalloproteinase, as well as IL-1, TNF-α, and COX-2, to reduce the inflammation in synovial cartilage. A recent study of 250 patients with cervical and lumbar disc disease, who were taking NSAIDs, revealed that 59% could substitute fish oil supplements as a natural anti-inflammatory agent for the NSAIDs.[71] The recommended dosage is a total of 1.5–5g of EPA and DHA per day, taken with meals.

Rare side effects include steatorrhea and occasional belching if the supplements are not taken with meals. Typically, persons on a regimen of anticoagulant medications should not take omega-3 EFAs because of the possibility of increasing the bleeding potential.

### White willow bark

Bark from the white willow tree is one of the oldest herbal remedies for pain and inflammation, dating back to ancient Egyptian, Roman, Greek, and Indian civilizations, as an analgesic and antipyretic agent. Because of the gastric side effects of aspirin, there has been a resurgence in the use of white willow bark for the treatment of inflammatory syndromes. The mechanism of action of white willow bark is similar to that of aspirin which is a nonselective inhibitor of COX-1 and COX-2, used to block inflammatory prostaglandins.[48]

Various randomized, placebo-controlled studies comparing white willow bark with nonsteroidal agents have shown an efficacy comparable to these agents and aspirin. Salicin from white willow bark is converted to salicylic acid by the liver and is considered to have fewer side effects than aspirin. However, it is costlier than aspirin, and should not be used in children (to avoid the risk of Reye’s syndrome), or in patients with peptic ulcer disease, poorly controlled diabetes, hepatic or renal disorders, or other conditions in which aspirin would be contraindicated. The usual dose of white willow bark is 240 mg/day.[18,19,33,41,64,69,99,100]

### Curcumin (turmeric)

Curcumin is a naturally occurring yellow pigment derived from turmeric (*Curcuma longa*), a flowering plant of the ginger family. It has traditionally been used as a coloring and flavoring spice in food products. Curcumin has long been used in both Ayurvedic and Chinese medicines as an anti-inflammatory agent, a treatment for digestive disorders, and to enhance wound healing. Several clinical trials have demonstrated curcumin’s antioxidant, anti-inflammatory, and antineoplastic effects. Results of a study by Zandi and Karin suggested that curcumin might be efficacious in the treatment of cystic fibrosis because of its anti-inflammatory effect.[121] Curcumin is known to inhibit inflammation by suppressing NF-kB, restricting various activators of NF-kB as well as stemming its expression.

Curcumin has also been suggested as a treatment for colitis, chronic neurodegenerative diseases, arthritis, and cancer. In addition, it regulates the activity of several enzymes and cytokines by inhibiting both COX-1 and COX-2. Most studies to date have been performed in animals, but given the centuries of use of curcumin, as well as its now demonstrated activity in the NF-kB, COX-1, and COX-2 inflammatory pathways, it may be considered a viable natural alternative to nonsteroidal agents for the treatment of inflammation.

The usual dosage of standardized turmeric powder is 400–600 mg taken three times per day.[13] Side effects are few, but with extended use, this agent can cause stomach upset, and in extreme cases gastric ulcers may occur at very high doses. Caution should be used if the patient is taking anticoagulant medications or high doses of nonsteroidal drugs. Studies have shown that curcumin may be used in combination with lower doses of nonsteroidal medications.[7–9,11,21,40,87,111,121]

### Green tea

Green tea has long been recognized to have cardiovascular and cancer preventative characteristics due to its antioxidant properties. Its use in the treatment of arthritic disease as an anti-inflammatory agent has been recognized more recently. The constituents of green tea are polyphenolic compounds called catechins, and epigallocatechin-3 galate is the most abundant catechin in green tea.

Epigallocatechin-3 galate inhibits IL-1–induced proteoglycan release and type 2 collagen degradation in cartilage explants.[44] In human *in vitro* models, it also suppresses IL-1b and attenuates activation of the transcription factor NF-kB. Green tea also inhibits the aggrecanases which degrade cartilage.

Green tea research now demonstrates both anti-inflammatory and chondroprotective effects. Additionally, green tea research includes the “Asian paradox”, which theorizes that increased green tea consumption in Asia may lead to significant cardiovascular, neuroprotective and cancer prevention properties.[113] The usual recommendation is 3–4 cups of tea a day. Green tea extract has a typical dosage of 300–400 mg. Green tea can cause stomach irritation in some, and because of its caffeine content, a decaffeinated variety is also available; but the polyphenol content is currently unknown.[2,49,53,108,112,117,120]

### Pycnogenol (maritime pine bark)

Pycnogenol, like white willow bark, is a nutraceutical material that has been used since ancient times. Pycnogenol is derived from the bark of the maritime pine tree (*Pinus maritima*) and has been used for more than 2000 years. It has been considered helpful for wound healing, treating scurvy, healing of ulcers, and reducing vascular inflammation. It contains a potent blend of active polyphenols, which includes catechin, taxifolin, procyanidins, and phenolic acids. It is one of the most potent antioxidant compounds currently known.[17,118]

Pycnogenol inhibits TNF-α–induced NF-kB activation as well as adhesion molecule expression in the endothelium. Grimm *et al*, recently reported that oral intake of pycnogenol inhibited NF-kB activation in lipopolysaccharide-stimulated monocytes as well, thus decreasing the inflammatory response. It also statistically significantly inhibited matrix metalloproteinase-9.[46] This matrix-degrading enzyme is highly expressed at sites of inflammation, and contributes to the pathogenesis of various chronic inflammatory diseases.[96]

Studies have shown that pycnogenol is 50–100 times more potent than vitamin E in neutralizing free radicals and that it helps to recycle and prolong the activity of vitamins C and E. Studies have shown pycnogenol to be effective in reducing blood pressure and reducing the risk of venous thrombosis by its effect on vascular endothelium. The usual dosage is 100–200 mg daily. Few side effects from the use of pine bark extracts have been reported, the most frequent being mild gastrointestinal effects such as diarrhea and upset stomach. Pycnogenol should not be taken by patients who are being treated with immunosuppressants or by those receiving corticosteroid drugs because it can enhance immune system function and interact with drugs that suppress the immune system.[46–84]

### Boswellia serrata *resin (Frankincense)*

The *Boswellia* species are trees located in India, Ethiopia, Somalia, and the Arabian Peninsula, and they produce a gum resin called olibanum, better known in the western world as frankincense. This resin possesses anti-inflammatory, anti-arthritic, and analgesic properties. *Boswellia* can inhibit the leukotriene biosynthesis in neutrophilic granulocytes by inhibiting 5-LOX, thus affecting various inflammatory diseases that are perpetuated by leukotrienes.[95] Clinically, the substance is used in the treatment of degenerative and inflammatory joint disorders. It reduces the total white blood cell count in joint fluid, and it also inhibits leukocyte elastase, which is released in rheumatoid arthritis. In one recent study, a statistically significant improvement in arthritis of the knee was shown after 8 weeks of treatment with 333 mg *B. serrata* extract taken three times a day. The treatment improved function, but radiographically there was no change in the affected joints.[62]

A combination of *Boswellia* and curcumin showed superior efficacy and tolerability compared with nonsteroidal diclofenac for treating active osteoarthritis. *Boswellia* typically is given as an extract standardized to contain 30-40% boswellic acids (300-500 mg two or three times/day). *Boswellia* has been well tolerated in most studies, although some people may experience stomach discomfort, including nausea, acid reflux, or diarrhea.[1–10,42,48,56,62,103,104]

### Resveratrol

Resveratrol is a plant-based polyphenol molecule that is found in various concentrations of many different plant sources. The plant is called Japanese Knot weed or *Polygonum cuspidatum*, and the skins of red wine grapes are believe to have the most concentrated amounts of resveratrol. In plants, resveratrol is generally found in the plant skin and acts as a phytoalexin to protect the plant from infection, excessive UV radiation and aide in general plant defense. Resveratrol has also been found to have significant anti-mutation, anti-inflammatory, antoxidant and DNA protective actions, when consumed by animals and humans.

Most of the active research with resveratrol has been done in neuro and cardioprotection, but several studies are being reported on resveratrol’s use for arthritic joint pain. Elmali *et al*, reported in 2007 using animals that intra-articular injection of resveratrol protects cartilage and reduces the inflammatory reaction in simulated knee osteoarthritis. The anti-inflammatory properties of resveratrol have also been observed in experimental animal models with paw edema, which is attributed to suppression of inflammatory prostaglandin synthesis.[29] Resveratrol is also a potent and specific inhibitor of TNF-α- and IL-1b-induced NF-kB activation. Resveratrol shows the anti-inflammatory properties as it suppresses COX-2 by blocking NF-kB activation.

Resveratrol is available commercially as a dietary supplement capsule, generally from the *P. cuspidatum* source. The *trans*-resveratrol is the active form, and although there is not an established dosing range, the typical dose is from 50 to 500 mg daily. Any significant side effect or safety issues with resveratrol have not been established, but due to an experimentally shown anti-platelet effect, caution should be exercised when taking other prescription or herbal anti-platelet or coagulation altering products.[29,54,59,68,72,107,109]

### Uncaria tomentosa *(cat’s claw)*

*Uncaria tomentosa* and *Uncaria guianensis* are Peruvian herbs derived from woody vines with small claw-like thorns (hence the vernacular name, cat’s claw) at the base of the leaf, which allow the plant to climb to heights of up to 100 ft. Traditionally, the bark of cat’s claw is used to treat arthritis, bursitis, and intestinal disorders. The active ingredients appear to be polyphenols (flavonoids, proanthocyanidins, and tannins), alkaloids, and sterols. Various studies indicate that this Peruvian herb induces a generalized reduction in proinflammatory mediators.

This herb has been shown to prevent the activation of the transcriptional factor NF-kB and it directly inhibits TNF-α production by up to 65-85%. It inhibits the expression of inducible genes associated with inflammation, specifically negating the expression of inducible nitric oxide synthase, and hence attenuates nitrous oxide production. Side effects may include nausea, although it has shown an impressive protective effect on indomethacin-induced enteritis in laboratory studies.

In general, toxicity and side effects are considered minimal. Two case reports of acute renal failure in a patient with lupus erythematosus have been recorded. Cat’s claw can be consumed as a tea (1000 mg root bark to 8 oz water), or as a dry, standardized extract in a capsule (20-60 mg daily).[37–39,76,86,97,98]

### Capsaicin (chili pepper)

*Capsicum annum* is a small spreading shrub which was originally cultivated in the tropical regions of the Americas but is now grown throughout the world, including the US. The small red fruit commonly used to accentuate chili owes its stinging pungency to the chemical, capsaicin. This was isolated by chemists more than a century ago and constitutes approximately 12% of the chili pepper. This fruit has been used for various medicinal purposes by the native peoples of the American tropics for hundreds of years.

Capsaicin produces highly selective regional anesthesia by causing degeneration of capsaicin-sensitive nociceptive nerve endings which can produce significant and long-lasting increases in nociceptive thresholds. Capsaicin potently activates transient receptor potential vanilloid 1, which is a main receptor underlying nociception. It also inhibits NF-kB, thus producing an anti-inflammatory effect. Capsaicin can cause a burning sensation when it comes in contact with human flesh, and also in the digestive tract. This herb is rarely used alone but is generally mixed into other natural anti-arthritic preparations. There are topical capsaicin formulations now available to treat post-herpetic neuralgia. Other uses have been studied for peripheral neuropathies and chronic musculoskeletal pain.[15,20,35,55,58,88,110]

## CONCLUSIONS

The human body’s natural response to injury results in inflammation-induced pain, swelling, and erythema. In order to reduce pain, anti-inflammatory agents such as NSAIDs act on the multiple inflammatory pathways, which, although often very effective, can have undesirable side effects such as gastric ulceration and, infrequently, myocardial infarction and stroke.

For centuries, natural anti-inflammatory compounds have been used to mediate the inflammatory process and often with fewer side effects. We have briefly reviewed several of the most commonly used plant- and animal-derived natural compounds that may possess similar effectiveness in treating the inflammatory reaction seen in both chronic and sub-acute pain syndromes encountered in a typical neurosurgical practice. Ongoing experiments and clinical trials should be continued to guide and provide their scientifically based effectiveness to reduce inflammation and promote wellness.

### Disclosures

Author #1 is the Chairman of Medical Advisory Board for GNC and a Shareholder of Herbals USA. Author #2 is a Shareholder of Herbals USA.
